# Accuracy of machine learning models for mitral regurgitation severity assessment: A systematic review and meta-analysis

**DOI:** 10.1016/j.ijcrp.2026.200642

**Published:** 2026-04-22

**Authors:** Pooya Eini, Golnaz Houshmand, Homa serpoush, Mohammad Rezayee, Milan Kassulke

**Affiliations:** a Cardiovascular Research Center, Rajaie Cardiovascular Institute, Tehran, Iran; b Cardiovascular Imaging Research Center, Rajaie Cardiovascular Institute, Tehran, Iran; c Hamadan University of Medical Sciences, Hamadan, Iran; d College of Human Medicine, Michigan State University, East Lansing, MI, USA

**Keywords:** Mitral regurgitation, Machine learning, Deep learning, Valvular heart disease, Cardiac imaging

## Abstract

**Background:**

Accurate assessment of mitral regurgitation (MR) severity is crucial for guiding clinical management, but is often limited by the subjectivity and variability of traditional echocardiographic evaluations. Machine learning (ML) models offer potential for automated, objective MR grading, yet their diagnostic performance remains underexplored. This systematic review and meta-analysis aim to evaluate the diagnostic accuracy of ML-based models for assessing MR severity.

**Methods:**

We searched five different databases for studies evaluating ML algorithms (deep learning or traditional ML) for MR severity assessment in adults. Data were extracted and the risk of bias was assessed using the PROBAST + AI tool. A bivariate random-effects model was used to pool diagnostic metrics, with heterogeneity quantified via I^2^ statistics and explored through meta-regression and subgroup analyses. Publication bias was evaluated using Deeks’ test and funnel plot.

**Results:**

Nine studies met inclusion criteria, demonstrating strong ML performance with a pooled AUROC of 0.97 (95% CI: 0.96–0.98), sensitivity of 0.93 (95% CI: 0.83–0.97), and specificity of 0.96 (95% CI: 0.92–0.98). High heterogeneity (I^2^ > 70%) was observed, partly explained by variations in validation methods and sample size. No significant publication bias was detected (Deeks’ p = 0.64). The certainty of the evidence was moderate due to heterogeneity and the retrospective study design.

**Conclusion:**

ML models demonstrate good diagnostic accuracy for assessing MR severity, with the potential to enhance clinical decision-making by reducing subjectivity. However, high heterogeneity and limited external validation necessitate prospective, standardized trials to ensure generalizability and clinical adoption.

## Highlights

• ML models show high accuracy for mitral regurgitation severity, with pooled AUROC 0.97 and sensitivity/specificity 0.96.
• Across nine studies, ML methods consistently outperformed conventional echocardiographic interpretation.
• Substantial heterogeneity was observed, partly due to differences in validation strategies and dataset sizes.
• Prospective studies with standardized methods and external validation are needed before clinical use.

## 1 Introduction

Mitral regurgitation (MR) stands out as the leading valvular heart condition, affecting around 1.7% of adults with notable clinical impact and reaching an estimated 24 million individuals globally [1,2]. Its occurrence escalates with advancing age, rising to 9.3% among those aged 75 and older [2]. Estimates from population-based research indicate that mild (grade I) MR affects 19.2% of individuals, while moderate (grade II), moderate-to-severe (grade III), and severe (grade IV) forms occur in 1.6%, 0.3%, and 0.2% of cases, respectively [3]. A substantial portion—over 50%—of valvular heart diseases, including MR, often goes undetected [4]. Individuals with MR face elevated risks of death and diminished well-being, with MR-related fatalities showing an upward trend in recent years [5]. Greater MR severity correlates directly with higher mortality rates, particularly in moderate and severe cases when compared to unaffected peers [6]. Those with mild MR may remain without symptoms [7]. In contrast, severe MR can lead to complications like elevated pulmonary wedge pressure, irregular heart rhythms, ventricular dysfunction, or fatal outcomes [8]. Thus, precise determination of MR extent plays a pivotal role in guiding diagnosis, therapeutic choices, and long-term predictions [9]. With emerging interventions for advanced MR, the demand for reliable identification has grown even more pressing [10].

Artificial intelligence (AI) refers to computer methods that mimic human thinking and decision-making [11]. Within this domain, deep learning models (DL) characterized by layered processing networks, proves especially effective for analyzing visual and health-related datasets [12]. When applied to extensive, detailed datasets for targeted applications, DL systems can be more accurate than experienced experts [13]. Emerging research underscores the value of AI and machine learning (ML) in heart imaging techniques [14]. In echocardiography, innovative AI solutions are advancing capabilities for capturing images and automatically measuring heart chamber dimensions and performance [15]. Such innovations could address longstanding issues like inconsistencies between and within observers during ultrasound evaluations [16]. By doing so, they promote greater reliability, speed, and uniformity in diagnostics [17]. That said, the use of DL for evaluating flow-based abnormalities via color Doppler, such as in MR, has received limited attention and poses greater technical hurdles compared to earlier AI achievements in echocardiography [18]. Moreover, prior AI-driven analyses of MR through echocardiography have mostly depended on visual traits, isolated metrics, or qualitative indicators [19].

Accurate classification of MR intensity is vital, given its links to substantial health burdens and death rates in more advanced stages [1]. Developing automated systems to detect high-risk MR could help doctors recognize it sooner, improve diagnostic accuracy and guide care more effectively [8]. This systematic review and meta-analysis seeks to consolidate existing data on ML innovations and their utility in evaluating MR severity risks, tackling echocardiography-related obstacles like observer discrepancies and the pursuit of consistent quantitative evaluations.

## 2 Methods

### 2.1 Study design and registration

We conducted a systematic review and meta-analysis of diagnostic test accuracy (DTA) in accordance with the Preferred Reporting Items for Systematic Reviews and Meta-Analyses (PRISMA) guidelines, which provide a structured framework for transparent reporting of DTA reviews to enhance reproducibility and minimize reporting biases [20]. The study protocol was prospectively registered in PROSPERO (registration number: CRD420251137460).

### 2.2 Search strategy

A comprehensive literature search was performed across multiple electronic databases—PubMed, Embase, Web of Science, Scopus, and EBSCO—from their inception to August 10, 2025, to capture all relevant publications up to the current date. Although the original PROSPERO protocol listed CINAHL and SCI, these were supplemented (not replaced) with EBSCO and Web of Science to ensure comprehensive coverage of grey literature and recent conference proceedings. The search strategy integrated controlled vocabulary terms (MeSH terms in PubMed and Emtree terms in Embase) with free-text keywords related to “mitral regurgitation,” “MR severity,” “artificial intelligence,” “machine learning,” “deep learning,” and “neural networks”. This combination was designed to maximize sensitivity while maintaining specificity, using Boolean operators (AND/OR) to link concepts. No language restrictions were imposed to avoid language bias. The full, reproducible search strings for each database, including any filters or limits applied, are detailed in Supplementary Table 1.

### 2.3 Eligibility criteria

Studies were eligible for inclusion if they met the following predefined criteria, aligned with the PICOS (Population, Index test, Comparator/reference standard, Outcome, Study design) framework for DTA reviews:

Population: Adult patients (≥18 years) undergoing assessment for mitral regurgitation (MR) severity, encompassing both primary (degenerative) and secondary (functional) MR etiologies, as well as mixed populations.

Index Test: AI-based algorithms (including machine learning or deep learning models) for automated or semi-automated assessment of MR severity, typically classified as mild, moderate, or severe based on quantitative or qualitative metrics.

Reference Standard: Established clinical criteria for MR severity, such as those from the American Society of Echocardiography (ASE) or European Association of Cardiovascular Imaging (EACVI), using imaging modalities like echocardiography (transthoracic or transesophageal) or cardiac magnetic resonance imaging (MRI) with confirmed diagnostic confirmation.

Outcomes: Studies were eligible if they reported diagnostic accuracy metrics (sensitivity, specificity, AUROC, or confusion matrices) for machine-learning models classifying MR severity on echocardiographic images or videos. Studies whose primary focus was phenogrouping or prognostic stratification were included only if they also provided extractable data on diagnostic classification of MR severity. Upon re-evaluation, all included studies met these criteria, as phenogrouping approaches were used to support or directly inform severity grading and diagnostic performance metrics were explicitly reported.

Study Design: Original research with validation components (internal, external, temporal, or cross-validation) to evaluate generalizability, excluding purely developmental studies without performance assessment.

Exclusion criteria included animal or in vitro studies, case reports/series (n < 10), reviews/editorials, studies lacking a clear reference standard or with non-standard MR definitions, those without extractable quantitative data, or duplicates.

### 2.4 Data extraction

Data extraction was performed in duplicate by two independent reviewers using a piloted, standardized electronic form developed in Microsoft Excel to ensure consistency and reduce errors. Extracted items encompassed:

Study characteristics (design, country/setting, publication year).

Model details (AI type; input features like Doppler parameters or image segments; development/validation methods).

Participant demographics (sample size for training/validation cohorts, mean age, sex distribution, MR etiology, comorbidities).

Performance metrics (AUROC, sensitivity/specificity, F1 score, Precision).

Additional elements (imaging modality, handling of missing data).

Discrepancies were resolved via consensus or consultation with a third reviewer. For studies reporting multiple models or thresholds, we prioritized the primary or best-performing model as specified by the authors.

### 2.5 Definition of target condition and construction of 2 × 2 tables

The target condition was defined as severe MR versus non-severe MR (mild or moderate), consistent with the most common clinical threshold used for intervention decision-making. When studies reported ordinal grading (mild/moderate/severe), moderate-to-severe MR was also analyzed as a secondary threshold. For multiclass outputs, the highest probability class was used to construct 2 × 2 contingency tables. Studies reporting only continuous risk scores were dichotomised at the study-specific optimal threshold that maximised the Youden index. All 2 × 2 tables were reconstructed directly from reported sensitivity, specificity, and prevalence or from confusion matrices provided in the original publications.

When multiple machine-learning models or operating thresholds were reported within a single study, the primary model (as designated by the study authors) or the best-performing model (highest AUROC) was extracted. This approach was prespecified in the protocol.

### 2.6 Risk of bias and certainty assessment

Risk of bias (ROB) and applicability were assessed using the Prediction model Risk Of Bias Assessment Tool for Artificial Intelligence (PROBAST-AI) [21], which was chosen over the originally planned QUADAS-2 because it is specifically tailored to machine-learning diagnostic models and provides domain-specific signaling questions for participant selection, predictor selection, model development, and analysis. This amendment was documented prior to data extraction and is registered in the updated PROSPERO record.

The overall certainty of evidence was appraised using the Grading of Recommendations Assessment, Development, and Evaluation (GRADE) approach adapted for DTA, starting at high certainty and downgrading for RoB, inconsistency, indirectness, imprecision and publication bias. Assessments were conducted independently by two reviewers, with discrepancies resolved by consensus.

### 2.7 Statistical analysis

All analyses were conducted in R version 4.5.0 using specialized packages for DTA meta-analysis, including mada for bivariate modeling and metafor for general random-effects meta-regression. A bivariate random-effects model was applied to jointly pool sensitivity and specificity, accounting for their inherent negative correlation and threshold variability across studies. Heterogeneity was quantified separately for sensitivity, specificity, and DOR using the I^2^ statistic (with values greater than 50% indicating substantial heterogeneity) and tau [2] for between-study variance. Sources of heterogeneity were explored through pre-specified subgroup analyses stratified by algorithm type (deep learning vs. traditional machine learning), imaging modality and input type (image vs video), MR assessment strategy (between studies using quantitative Doppler based MR reference standards and those relying on non-quantitative, algorithmic, or expert based classification) and studies used traditional echocardiographic assessment (human interpretation or guideline-based measurements • PISA, VC, RF, RVol • ASE/ACC guideline grading) with machine-learning–based analysis. Meta-regression incorporated these moderators to quantify their impact on effect sizes, using restricted maximum likelihood estimation.

Publication bias and small-study effects were assessed using Deeks' funnel plot asymmetry test, specifically designed for DTA meta-analyses to detect if smaller studies disproportionately report higher accuracy. A p-value <0.10 was considered indicative of potential bias. Sensitivity analyses, including leave-one-out, were performed to evaluate the robustness of the results. All statistical tests were two-sided with α = 0.05, and forest plots/HSROC curves were generated for visual interpretation.

## 3 Results

### 3.1 Study selection

**Fig. 1:**
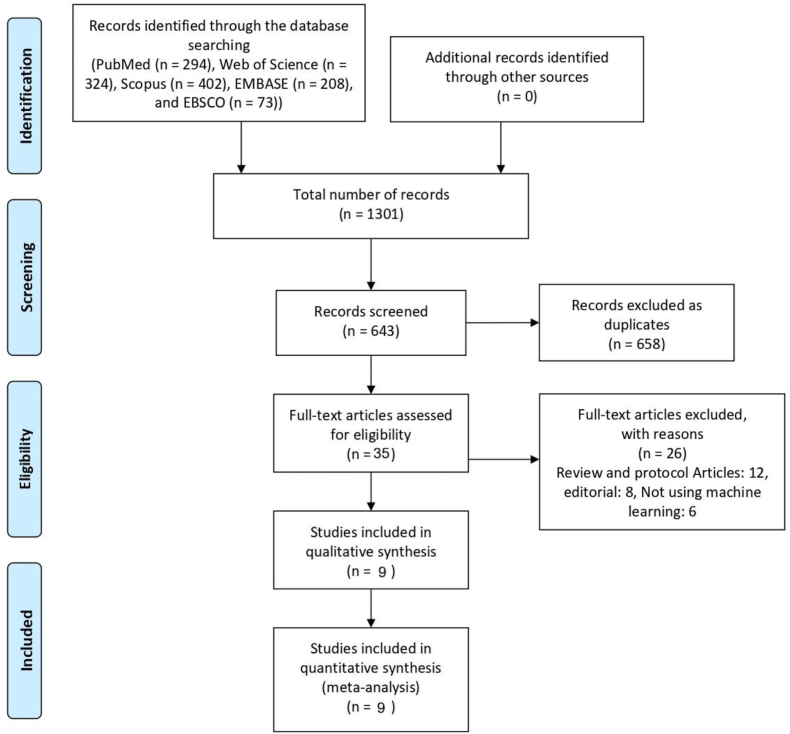
PRISMA flowchart for study selection.

A comprehensive literature search was conducted across five electronic databases: PubMed (n = 294), Web of Science (n = 324), Scopus (n = 402), EMBASE (n = 208), and EBSCO (n = 73), yielding a total of 1301 records. After removing 658 duplicates, 643 unique articles remained for title and abstract screening. Of these, 35 studies were selected for full-text review, resulting in the inclusion of 9 studies that met the predefined eligibility criteria. Fig. 1 shows the PRISMA flowchart for study selection.

### 3.2 Studies characteristics

This systematic review and meta-analysis synthesized data from 9 retrospective observational studies published between 2016 and 2025. These studies collectively represent a global effort to leverage computational approaches for enhancing echocardiographic or magnetic resonance-based assessments, with a primary emphasis on automating quantitative or qualitative MR classification. Geographically, the investigations were distributed across Asia (Iran: n = 1; China: n = 3; India: n = 1), North America (USA: n = 2; Canada, in collaboration with France: n = 1), and Europe (France, in collaboration with Canada: n = 1), alongside one multicenter study involving institutions from the USA, Singapore, Sweden, and other regions. This distribution highlights a predominance of contributions from Asian research centers (60%).

All included studies were retrospective in design, drawing on archival imaging datasets without prospective enrollment or intervention arms. Validation strategies varied: four relied solely on internal validation (cross-validation within a single dataset), while the remaining five incorporated both internal and external validation, often using independent cohorts from different institutions or countries to assess generalizability. This mixed approach underscores efforts to mitigate overfitting, though the absence of prospective external validation in all studies limits inferences about real-world clinical deployment (see Table 1).

Imaging modalities were 2D echocardiography videos, color Doppler echocardiography, transthoracic echocardiography (TTE) with color Doppler, and multiparametric echocardiography. Feature selection was heterogeneous, reflecting the multifaceted nature of MR quantification. Common inputs included textural descriptors (Extensive Local Binary Patterns [ELBP] and wavelet-based features), geometric and flow parameters (effective regurgitant orifice area [EROA], vena contracta [VC], regurgitant volume [RV], proximal isovelocity surface area [PISA] radius), and segmentation-derived metrics (MR jet area, left atrium area). The most influential features, as identified through model interpretability or performance metrics, often centered on flow convergence regions (radius for EROA/RV computation), texture patterns from Daubechies wavelets (db4), and integrated multiparametric sets (9-24 echocardiographic variables), emphasizing the value of multidimensional data fusion for robust MR grading. Detailed methodological characteristics of each study are summarized in Table 2.

**Table 1:** Included studies characteristics.

First author/Year	type of study	country	validation	selected features	most important features	sample size	mean age	male%	Follow up (Months)	Machine learning algorithm	Best algorithm
Moghaddasi/2016 [22]	Retrospective	Iran	Internal (5-fold cross-validation)	Extensive Local Binary Pattern (ELBP), Extensive Volume Local Binary Pattern (EVLBP), textural descriptors	Extensive Uniform Local Binary Pattern (ELBPU), Extensive Volume Local Binary Pattern (EVLBP)	139 (37 normal + 34 mild + 32 moderate + 36 severe)	NR	NR	NR	Support Vector Machine (SVM), Linear Discriminant Analysis (LDA), Template Matching	SVM
Balodi/2020 [23]	Retrospective	India	Internal (10-fold cross-validation)	Daubechies wavelet-based texture features (7 statistical features per decomposition level, up to 4 levels)	Texture features from db2, db4 wavelets	345 (140 mild + 140 moderate + 65 severe)	NR	NR	NR	Support Vector Machine (SVM)	SVM
Zhang/2021 [24]	Retrospective	China	Internal and external	MR types per 2017 ASE guidelines (grades I-IV), Mask R-CNN for segmentation	MR jet features, classification into 4 grades	1427 (370 grade I, 353 grade II, 344 grade III, and 360 grade IV)	68	35%	NR	Mask R-CNN algorithm	CNN
Yang/2022 [25]	Retrospective	China	Internal and external	MR jet area, left atrium (LA) area	MR jet and LA segmentation features	1480 (592 normal +462 mild + 300 moderate + 126 severe)	70	66%	NR	Self-supervised learning (SSL)	SSL
Bernard/2023 [26]	Retrospective	France, Canada	Internal and external (development cohort from France, validation from Canada)	24 echocardiographic parameters (effective regurgitant orifice (ERO), vena contracta (VC), regurgitant volume)	Not explicitly ranked; all 24 parameters integrated via unsupervised/supervised ML	400 (54 mild + 124 mild-to-moderate + 105 moderate-to-severe + 117 severe)	61	60%	Median 3.2 years (IQR 1.3-5.3) France; 6.8 years (IQR 4.0-8.5) Canada	Unsupervised clustering	Unsupervised clustering
Long/2024 [27]	Retrospective	USA	Internal and external	Color MR Doppler videos, 4-step and 6-step MR severity scales	Features from apical 4-chamber and multiple views	52,802 (33,735 normal + 11,592 mild + 4209 mild-moderate + 2087 moderate + 661 moderate-severe + 518 severe)	64.3 ± 16.5	NR	NR	Deep learning (DL)	Deep learning (DL)
Zhong/2024 [28]	Retrospective	China	Internal	MR flow convergence region, radius for effective regurgitant orifice area (EROA), regurgitant volume (RV)	Radius, EROA, RV	269 (50 grade I + 63 grade II + 66 grade III + 90 grade IV)	65	60	NR	Convolutional Neural Network (CCN)	Convolutional Neural Network (CCN)
Sadeghpour/2025 [29]	Retrospective	Multicenter	Internal and external	16 MR-related parameters	vena contracta from parasternal long axis, apical 2-chamber, and apical 4-chamber views; regurgitation area ratio from apical 2-chamber and apical 4-chamber views; continuous wave Doppler density; left ventricular end-diastolic volume; left ventricular outflow tract stroke volume; and pulmonary artery systolic pressure.	438 (29 none/trace + 151 mild + 92 moderate + 166 severe)	73	57.30%	12 months (1-year mortality)	Catboost	Catboost
Long/2025 [30]	Retrospective	USA	Internal and external	Color MR Doppler videos, regurgitation severity classification (4-step and 6-step scales)	Not explicitly ranked; features from multiple videos/views	46,658 (20,966 none + 15,607 mild + 5646 mild–moderate + 2831 moderate + 901 moderate–severe + 707 severe)	61.8	47.20%	NR	Deep learning (DL)	Deep learning (DL)

**Table 2:** Methodological characteristics of each study.

First author/Year	Imaging Modality	Equipment	Image Specifications	Echocardiographic Views	Data Handling	Synchronization	MR Severity Assessment	Additional Imaging	Experimental Frames for Analysis
**Moghaddasi/2016**	TTE	GE Vivid 7	480 × 640 px (DICOM); 40 fps; 1.7–3 MHz	A4C, A2C, PSAX	Offline blinded analysis on workstation	ECG-synchronized	ACC/AHA guideline grading by 2 readers; adjudication by 3rd	TEE for borderline cases	3 frames (early, mid, end-systole) via ECG
**Balodi/2020**	TTE (Color)	Not specified	800 × 600 px; RGB→grayscale	3 standard TTE views	ROI extraction → DWT→texture features→SVM (RBF) with 10-fold CV	NR	Expert-graded mild/moderate/severe	None	Image-based (not frame-based); Multiview majority voting
**Zhang/2021**	2D-TTE Color Doppler	Philips EPIQ 7C; GE Vivid E95; Siemens SC2000	VC, PISA (Nyquist 30–40 cm/s), RF (50–70 cm/s), RVol	A4C or best jet-visualizing view	Two-center dataset; quantitative Doppler measurements per ASE 2017	NR	ASE 2017 mild/mod/subdivided I–IV	Zoom-mode VC, optimized PISA	Not frame-based; quantitative Doppler acquisition
**Yang/2022**	TTE (Color Doppler)	NR	NR	Required A4C-CDI; test set required PLAX, A4C, others (truncated)	Retrospective dataset (n = 2766)	NR	Clinical mild/moderate/severe; quantitative SV method for test cohort	NR	Video-based; no systolic frame protocol
**Bernard/2023**	TTE (Comprehensive Doppler)	Commercial systems (models NR)	Includes PISA + Simpson volumetrics; specifics NR	ASE-recommended MR views	Prospective collection; retrospective analysis in echo core labs	NR	Integrative semiquantitative + quantitative (PISA/Simpson) per ASE	None outside echo	Not frame-based
**Long/2024**	TTE (Color Doppler)	Philips ie33/Epiq; analysis via Syngo (internal) and Xcelera (external)	exclusion if < 50 clips or <5 MR color clips	Full TTE set with MR color views	Structured fields + NLP extraction; >200 reports manually verified	NR	ASE-guided 6-step → collapsed to 4-step	None	Clip-based only; no systolic-frame extraction
**Zhong/2024**	TTE (Color Doppler + CW)	Philips EPIQ 7C; Philips CX50	DICOM; PISA NL 30–40 cm/s; 4–6 segments, 3–5 cycles each	A4C-MVCDFI (VC, PISA, jet)	5164 PISA frames screened → 4104 train/val + 1060 test	Implicit systolic PISA frame alignment	ASE 2017 (I–IV), quantitative EROA/RV	CW Doppler for Vmax, VTI	Frame-based: manual PISA-frame extraction per case
**Sadeghpour/2025**	TTE (Color, PW, CW)	Multiple cohorts; machine models NR	DICOM; all systolic frames analyzed; grayscale norm for CWDD	PLAX/A2C/A4C for VC; A2C/A4C for RAR; CW for MR jet	Phase 1: view classifier→ROI→CNN→expert correction. Phase 2: automated measurement→ML severity model	Implicit cardiac-cycle sync (all systolic frames)	Core-lab ASE grading; significant = moderate + severe	None	Frame-intensive: all systolic frames in all clips aggregated
**Long/2025**	TTE (Color Doppler)	Philips ie33/Epiq	No parameters; exclusion if < 50 videos	A4C for MR; multiview ingestion of all MR-relevant clips	Whole-study ingestion→CNN video-level scoring→Single-View, Top-3, or Deep-Set Transformer study-level diagnosis	NR	ASE 6-step → collapsed to 4-step; structured + NLP labels; >200 manual checks	None	Video-level (not frame-level); all Doppler clips consumed

Abbreviations.

TTE – Transthoracic echocardiography, A4C/A2C/PLAX/PSAX – Apical 4-chamber, Apical 2-chamber, Parasternal Long-Axis, Parasternal Short-Axis, CDI – Color Doppler Imaging, DWT – Discrete Wavelet Transform, ROI – Region of Interest, SVM (RBF) – Support Vector Machine with a Radial Basis Function kernel, CV – Cross-Validation, PISA – Proximal Isovelocity Surface Area, VC – Vena Contracta, RF – Regurgitant Fraction, RVol – Regurgitant Volume, SV method – Stroke Volume method, EROA – Effective Regurgitant Orifice Area, CW/PW Doppler – Continuous-Wave and Pulsed-Wave Doppler, CWDD – Continuous-Wave Doppler Density, MVCDFI – Mitral Valve Color Doppler Flow Imaging, ASE – American Society of Echocardiography, ACC/AHA – American College of Cardiology and American Heart Association, TEE – Transesophageal Echocardiography, CNN – Convolutional Neural Network, NLP – Natural Language Processing, NR – Not Reported.

Sample sizes exhibited substantial variability, ranging from small cohorts (139 patients) to large-scale datasets (up to 71,660 TTEs in deep learning validations), with a median of approximately 350 where reported (though exact totals were unspecified in three studies, likely due to frame- or video-level analyses rather than patient-level counts, with mean ages between 42 and 73 years and male proportions from 35% to 66%. Follow-up periods were reported in only two studies, ranging from 1 to 6.8 years. ML algorithms spanned traditional and advanced paradigms, including Support Vector Machines (SVM; n = 2, often with wavelet or radiomics features), deep learning (DL) systems (n = 3, end-to-end convolutional networks for video-level classification), fully convolutional neural networks (FCN; n = 1), self-supervised learning (SSL; n = 1), and hybrid unsupervised/supervised approaches with explainable AI (n = 1). Multiple models were tested in two studies. The best-performing algorithms were typically domain-specific adaptations, such as SVM with ELBP/EVLBP or db4 wavelets, DL for Multiview TTE integration, FCN for segmentation, SSL for jet area quantification, and phenogrouping with explainable AI.

### 3.3 Overall pooled diagnostic accuracy

**Fig. 2:**
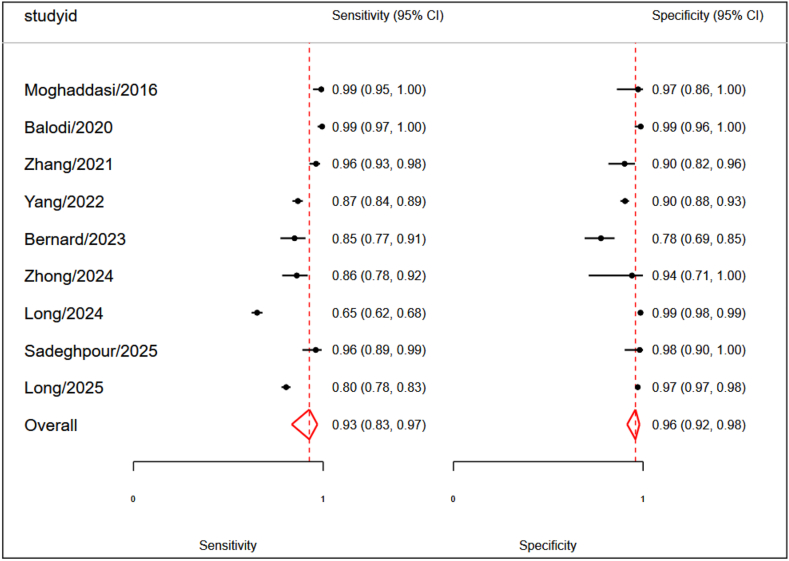
Forest plot for sensitivity and specificity.

**Fig. 3:**
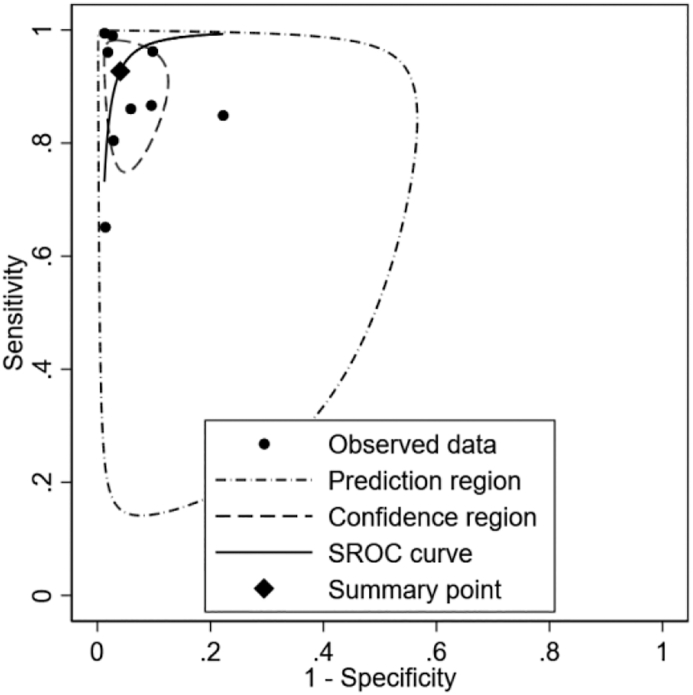
Summary receiver operating characteristics.

A meta-analysis of 9 studies evaluating AI algorithms for assessing MR severity demonstrated good overall diagnostic performance. For studies reporting multiple models or thresholds, we prioritized the primary or best-performing model as specified by the authors. The pooled sensitivity was 0.93 (95% CI: 0.83–0.97), indicating that AI algorithms correctly identified 93% of true positive MR cases. The pooled specificity was 0.96 (95% CI: 0.92–0.98), showing that 93% of true negative cases were correctly identified (Fig. 2). The pooled area under the receiver operating characteristic curve (AUROC) was 0.97 (95% CI: 0.96–0.98), reflecting excellent discriminatory ability (Fig. 3). Detailed performance metrics for different models across studies are provided in Table 3.

**Table 3:** Performance metrics across included studies.

First author/Year	algorithm	Accuracy	Sensitivity (Recall)	Specificity	Precision	F1score	AUROC
Moghaddasi/2016	Support Vector Machine (SVM)	0.99	0.99	0.97	0.99	NR	NR
	Linear Discriminant Analysis (LDA)	0.96	0.91	0.97	0.92	NR	NR
	Template Matching	0.96	0.93	0.97	0.91	NR	NR
Balodi/2020	Support Vector Machine (SVM)	0.99	0.99	0.99	0.99	NR	NR
Zhang/2021	Mask R-CNN algorithm	0.95	0.96	0.9	0.96	0.92	NR
Yang/2022	Self-supervised learning (SSL)	0.96	0.87	0.9	0.89	NR	0.95
Bernard/2023	Unsupervised clustering	0.81	0.85	0.78	0.8	0.814	0.829
Long/2024	Deep learning (DL)	0.94	0.65	0.99	0.88	0.79	0.98
Zhong/2024	Convolutional Neural Network (CCN)	0.91	0.86	0.94	0.86	0.81	NR
Sadeghpour/2025	Catboost	0.97	0.96	0.98	0.99	NR	NR
Long/2025	Deep learning (DL)	0.95	0.8	0.97	0.81	0.81	0.98

### 3.4 Heterogeneity assessment

Substantial statistical heterogeneity was observed across the included studies. The I^2^ statistic for sensitivity was 90.15%, and for specificity was 63.51%, both well above the conventional 50% threshold for high heterogeneity. This indicates that much of the variability in effect sizes likely reflects genuine differences between studies rather than sampling error alone.

### 3.5 Meta-regression analysis

Meta-regression analysis identified study sample size (log-transformed) as a significant source of heterogeneity (LRTChi [2] = 14.00, p < 0.001, I^2^ = 86%). Validation strategy also contributed (LRTChi [2] = 7.99, p = 0.02, I^2^ = 75%), with notable differences between internal and external validation groups. In contrast, model type (p = 0.08) and country of origin (p = 0.69) did not significantly explain heterogeneity (Supplementary Table 2).

### 3.6 Leave-one-out sensitivity analysis

A leave-one-out sensitivity analysis was conducted to assess the influence of individual studies on the pooled estimates. The results demonstrated remarkable stability in the pooled effect size across all iterations. When each study was sequentially omitted, the pooled effect size (log diagnostic odds ratio) ranged from 4.54 to 5.62, with the overall pooled estimate being 5.062 (95% CI: 3.176-6.948). This consistency indicates that no single study disproportionately influenced the overall findings (Supplementary Fig. 1).

### 3.7 Subgroup analysis

Among the included studies, traditional machine learning approaches such as SVM, CatBoost, semi-supervised learning, and unsupervised clustering were commonly applied. In contrast, deep learning methods, particularly convolutional neural networks (CNNs), were also frequently utilized, reflecting the growing trend toward DL-based architectures in MR severity assessment. Subgroup analyses demonstrated that traditional machine learning models (n = 4) achieved higher pooled sensitivity of 0.97 (95% CI: 0.90–0.99) and specificity of 0.96 (95% CI: 0.86–0.99), with moderate heterogeneity (I^2^ = 66.3% for sensitivity; I^2^ = 64.4% for specificity). In comparison, deep learning models (n = 5) showed lower pooled sensitivity of 0.85 (95% CI: 0.74–0.92) and specificity of 0.95 (95% CI: 0.90–0.98), accompanied by substantial heterogeneity (I^2^ = 94.8% and 61.6%, respectively) (Supplementary Fig. 2).

The subgroup analysis comparing studies that used individual frames versus those that used full video-based echocardiographic inputs demonstrated clear methodological performance differences. Frame-based models (n = 4) achieved very high pooled sensitivity (0.97, 95% CI 0.90–0.99) and specificity (0.98, 95% CI 0.94–0.99), with minimal between-study heterogeneity (I^2^: generalized = 0.00%, sensitivity = 66.27%, specificity = 13.11%). In contrast, video-based studies (n = 5) showed lower pooled sensitivity (0.85, 95% CI 0.74–0.92) but comparably high specificity (0.94, 95% CI 0.85–0.97), accompanied by substantial heterogeneity across all measures (I^2^: generalized = 89.58%, sensitivity = 94.85%, specificity = 89.72%). The corresponding forest plots for each subgroup are presented in Supplementary Fig. 3.

The subgroup analysis stratified by MR assessment strategy revealed differences in diagnostic performance and heterogeneity between studies using quantitative Doppler-based MR reference standards and those relying on non-quantitative, algorithmic, or expert-based classification. Studies using Doppler-quantified MR (n = 4) demonstrated a pooled sensitivity of 0.93 (95% CI 0.85–0.96) and specificity of 0.89 (95% CI 0.78–0.95), with low generalized heterogeneity (I^2^ = 0.01%) and moderate variability across sensitivity (76.48%) and specificity (60.64%). In contrast, studies employing non-quantitative or expert classification (n = 5) achieved a comparable pooled sensitivity of 0.94 (95% CI 0.73–0.99) and higher pooled specificity of 0.97 (95% CI 0.94–0.99), but at the cost of substantial heterogeneity (generalized I^2^ = 81.57%; sensitivity I^2^ = 92.12%; specificity I^2^ = 63.77%). The corresponding subgroup forest plots are presented in Supplementary Fig. 4.

The subgroup analysis comparing traditional echocardiographic assessment (n = 3) with machine-learning–based analysis (n = 6) demonstrated clear methodological effects on diagnostic performance and heterogeneity. Studies using conventional Doppler measurements or expert interpretation achieved a pooled sensitivity of 0.96 (95% CI 0.85–0.99) and specificity of 0.90 (95% CI 0.76–0.96), with minimal generalized heterogeneity (I^2^ = 0.14%) but moderate variability within sensitivity (78.87%) and specificity (73.35%). These studies predominantly relied on guideline-based human interpretation, incorporating parameters such as PISA, vena contracta, regurgitant fraction, and regurgitant volume. In contrast, ML/DL-based studies showed a pooled sensitivity of 0.91 (95% CI 0.76–0.97) and higher pooled specificity of 0.97 (95% CI 0.94–0.99), but with substantial heterogeneity (generalized I^2^ = 76.96%; sensitivity I^2^ = 93.21%; specificity I^2^ = 45.98%). Corresponding forest plots are provided in Supplementary Fig. 5.

### 3.8 Publication bias assessment

**Fig. 4:**
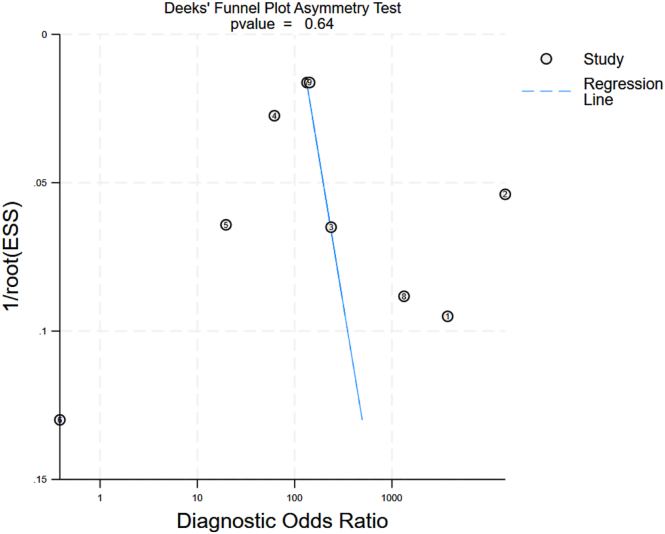
Deeks' Funnel plot asymmetry test.

Statistical tests for small-study effects and publication bias (Deek's test) yielded a p-value of 0.64, indicating no significant evidence of publication bias or asymmetry in the funnel plot. This suggests that the meta-analysis findings are unlikely to be influenced by the selective publication of studies with favorable results (Fig. 4).

### 3.9 Quality assessment

Overall, the included studies demonstrated varying levels of bias, with one study at low risk and the majority at moderate risk, primarily due to concerns in participant selection, outcome determination, and analytical rigor. For the participants' domain, most studies were rated as unclear or moderate risk. Zhang (2021) was unclear due to unreported consecutive recruitment, while Yang (2022) was unclear in development but moderate in evaluation, owing to a test set selected based on a reference standard, which potentially introduced selection bias. Bernard's 2023 study was unclear due to the heterogeneous cohorts from two countries, which lacked detailed spectral information. Moghaddasi (2016) was rated as having a moderate risk due to small sample sizes and a lack of representativeness.

**Fig. 5:**
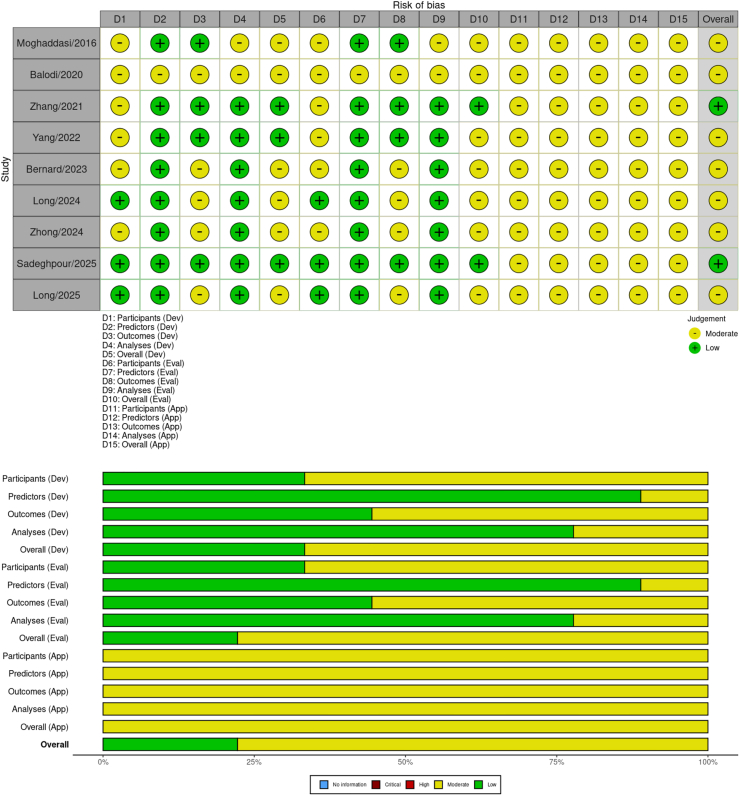
Risk of bias assessment using the PROBAST + AI tool.

In contrast, the predictors domain was consistently low risk across all studies, as predictors were derived from standard echocardiography or cardiac MRI data and clearly described, with no evidence of data leakage or overfitting in feature extraction. For the outcome's domain, ratings were mixed: Zhang (2021) and Yang (2022) were low risk, adhering to American Society of Echocardiography (ASE) guidelines with expert adjudication, while Bernard (2023) was moderate risk due to phenogroups potentially derived from predictors, raising concerns of circularity; Moghaddasi (2016) was low risk with guideline-based expert assessments. Long (2024) and Long (2025) were considered to have a moderate risk of outcomes due to their reliance on subjective cardiologist interpretations as the reference standard. Balodi (2020) and Zhong (2024) also reported moderate risk outcomes due to subjective grading without core lab verification. The analysis domain showed low risk in several studies, such as Zhang (2021) (external validation on a large sample), Yang (2022) (cross-validation with an external test set), and Bernard (2023) (SHAP explainability and external cohort), but moderate risk in Moghaddasi (2016), Balodi (2020), and Zhong (2024) due to small samples, internal validation only, and absence of external testing, increasing overfitting risks. Sadeghpour (2025) was associated with a low risk across domains, benefiting from multicenter data, automated parameters, core lab outcomes, and independent validation. The applicability to clinical practice was moderate in all studies, as none reported real-world deployment or integration into clinical workflows, highlighting a significant gap in translating AI models to clinical practice (Fig. 5). Supplementary Table 3 lists quality assessment check list based on PROBAT + AI tool.

### 3.10 GRADE assessment

The certainty of evidence was graded using the GRADE framework adapted for diagnostic accuracy studies. Starting from a high certainty, we downgraded to moderate overall due to moderate risk of bias in most studies (affecting consistency and precision), high inconsistency (I^2^ > 70% for sensitivity and specificity), and some indirectness (limited external validation in diverse populations). No downgrading occurred for imprecision (pooled estimates had reasonable CIs) or publication bias (non-significant Deek's test). This moderate certainty suggests that AI algorithms likely offer high diagnostic accuracy for MR severity, but further high-quality studies with objective outcomes and real-world validation are needed to strengthen confidence.

## 4 Discussion

This meta-analysis revealed that ML models demonstrate good diagnostic accuracy for assessing mitral regurgitation severity. Despite clinical and methodological heterogeneity (I^2^ > 70%), meta-analysis was deemed appropriate because all studies shared the common aim of evaluating machine-learning models for diagnostic classification of MR severity using echocardiographic data. Pooling across heterogeneous studies provides a broader overview of the field while acknowledging that results should be interpreted cautiously in light of these differences. However, high heterogeneity and reliance on retrospective designs highlight challenges to generalizability, necessitating cautious interpretation and further validation in diverse clinical settings. Given the limited number of studies (n = 9), meta-regression and subgroup analyses are exploratory and should be interpreted with caution owing to low statistical power and risk of unstable estimates.

### 4.1 Comparison of different machine learning models

The studies encompassed a spectrum of ML paradigms, reflecting the evolution from feature-engineered traditional ML to data-driven DL, tailored to the complexities of MR imaging data. Early investigations predominantly employed SVMs, a supervised ML algorithm effective for classification tasks with high-dimensional features. SVM was utilized in two studies to discriminate MR severity levels based on textural or radiomics features extracted from echocardiography or CMR images [22,23]. In Moghaddasi et al. study, SVM was combined with extensive local binary patterns (ELBP) and volume local binary patterns (EVLBP) to capture micro-patterns in 2D echocardiography videos, achieving accuracies of 99.52% for mild MR detection [22]. Similarly, Balodi et al. leveraged Daubechies wavelet decomposition for texture feature extraction, with SVM yielding superior performance using db4 wavelets across apical and parasternal views [23]. These SVM-based methods emphasize manual feature engineering, such as gradient differences or wavelet statistics, which are computationally efficient but reliant on domain expertise for feature selection.

In contrast, more recent studies shifted toward DL architectures, which automate feature extraction through convolutional layers, enabling end-to-end processing of raw imaging data. Convolutional neural networks (CNNs) and their variants dominated, appearing in five studies focused on segmentation and classification of color Doppler flows [[24], [25], [27], [28], [30]]. Zhang et al. applied the Mask R-CNN algorithm for segmenting MR jets in color Doppler images, classifying severity per American Society of Echocardiography (ASE) guidelines with accuracies of 0.90-0.91 across mild, moderate, and severe categories [24]. Zhong et al. utilized a fully convolutional neural network (FCN) to segment flow convergence regions and compute effective regurgitant orifice area (EROA) and regurgitant volume (RV), achieving grading accuracies of 0.88-0.95 in apical four-chamber views [28]. Self-supervised learning (SSL), a DL variant minimizing labeled data needs, was explored in Yang et al. study, where it segmented MR jet and left atrial areas, improving Dice similarity coefficients by 6.2-8.1% over residual U-Net baselines [25].

Hybrid approaches integrating unsupervised and supervised ML were less common but innovative for phenotyping and risk stratification. Bernard et al. employed unsupervised clustering followed by supervised ML and explainable artificial intelligence to integrate 24 echocardiographic parameters, delineating high- and low-severity MR phenogroups with incremental prognostic value over conventional profiles [26]. Similarly, Sadeghpour et al. developed multiple ML models to optimize nine parameters from 16 MR-related metrics, using ensemble techniques for severity grading with 0.80 accuracy across none-to-severe scales [29]. Other studies used advanced multiview DL systems for transthoracic echocardiography (TTE) videos, supporting both 4-step and 6-step ordinal classifications, with weighted kappa values of 0.84 and 0.80 in internal and external tests, respectively [27,30].

This diversity underscores a progression toward DL for handling volumetric and temporal data in echocardiography, offering scalability for large datasets (>70,000 TTEs [30]). However, traditional ML like SVM remains viable for smaller cohorts or when interpretability is prioritized, as in radiomics models.

### 4.2 Conventional methods compared to machine learning

ML models consistently outperformed or matched traditional qualitative or semi-quantitative methods, such as manual vena contracta (VC) measurement or proximal isovelocity surface area (PISA) calculations, in terms of efficiency, reproducibility, and prognostic utility. Traditional approaches, reliant on guideline-based multiparametric integration (e.g., 2017 ASE criteria), suffer from subjectivity and interobserver discordance, with reported variability up to 20-30% in MR grading. In contrast, ML models automated these processes, reducing analysis time to seconds (80 s per case [29]) while achieving high sensitivities (0.96) and specificities (0.98) for significant MR detection.

Direct comparisons with physicians highlighted ML's potential to augment clinical workflows. Zhong et al. demonstrated that FCN-based grading accuracies (0.82-0.90 across etiologies) surpassed junior physicians (with <5 years' experience) and approached senior experts, particularly in functional and degenerative MR subtypes [28]. Yang et al. reported that SSL-assisted grading improved physician sensitivity from 77.0% to 86.7% for moderate-to-severe MR, with stable specificity (∼91%), underscoring AI's role in reducing underdiagnosis in borderline cases [25]. Bernard's phenogrouping model provided incremental value over conventional severe MR classifications, with Harrell's C-statistic improvements (P = 0.048) and net reclassification gains (P = 0.002), predicting better event-free survival post-surgery in high-severity groups [26].

In large-scale validations achieved substantial agreement with cardiologist interpretations (weighted kappa 0.73-0.81), with multiview integrations outperforming single-view analyses (82% vs. 80% accuracy) [27,30]. These surpassed traditional methods in handling eccentric jets or primary/secondary etiologies, though slight performance dips were noted in eccentric MR. on the other hand, Machine learning–graded severe mitral regurgitation was associated with higher 1-year mortality (adjusted HR: 5.20; 95% CI: 1.24–21.9; P = 0.025), offering a prognostic advantage over mild cases and refining manual assessments [29].

Despite these advantages, ML models are not infallible; misclassifications often occurred between none/trace and mild MR (63-66% of errors [27,30]), mirroring physician challenges in low-severity distinctions. External validations generally showed modest declines (kappa 0.80 external vs. 0.84 internal [27]), emphasizing the need for diverse training data to mitigate site-specific biases.

### 4.3 Clinical Implications of machine learning models in clinical practice

ML-models offer a paradigm shift by automating quantitative analysis of echocardiographic data, thereby enhancing diagnostic precision and reproducibility. Beyond diagnostics, the integration of ML into clinical practice holds promise for personalized medicine. Systems like DELINEATE-MR, which employ deep learning for echo tracking and MR evaluation, have been shown to mitigate variability by providing objective, reproducible metrics, aiding in patient selection for TMVR where precise MR quantification is critical for procedural success and long-term durability [27]. In resource-limited settings, these models could democratize access to expert-level assessments via cloud-based platforms or embedded echo software, enabling point-of-care decisions in primary care or telemedicine scenarios. Moreover, explainable AI variants allow clinicians to interrogate model decisions, fostering trust and facilitating shared decision-making with patients. Prognostic applications extend to predicting MR progression or repair failure; for example, ML models trained on preoperative data have forecasted recurrence rates after mitral valve repair with AUC of up to 0.92, informing surgical strategies and follow-up protocols [31]. However, real-world adoption requires addressing regulatory hurdles, such as obtaining FDA clearance for AI as a medical device, and ensuring seamless workflow integration to avoid disrupting clinical routines.

### 4.4 Limitations of the study

Several methodological and evidential limitations constrain this review. Foremost, the analysis was based on only 9 studies, a relatively small number that limits the generalizability of the pooled estimates. This paucity of studies reflects the nascent stage of AI applications in valvular imaging, potentially overlooking emerging methodologies or underrepresented regions. High heterogeneity was evident across key metrics, indicating substantial variability attributable to differences in study designs and patient cohorts. Furthermore, the lack of unified data gathering protocols, reference standards, and data preprocessing techniques hinders direct comparisons. Additionally, most studies lacked external validation in diverse populations, potentially overestimating performance in controlled settings and underrepresenting underrepresented groups. These limitations underscore the need for cautious extrapolation to clinical practice and highlight gaps in the evidence base that future research must address to improve the understanding of this condition.

## 5 Future perspectives

Prospective, multicenter trials should be prioritized to validate ML models in real-time clinical environments, incorporating diverse patient demographics to enhance equity and generalizability, potentially through global consortia [32]. Standardization of data acquisition and reporting, such as adopting unified datasets (via federated learning to preserve privacy) and adhering to frameworks for transparent model development, will mitigate heterogeneity and facilitate meta-analyses with greater precision [33]. The integration of multimodal data, fusing echocardiography with cardiac MRI, biomarkers (BNP levels), and electronic health records, could yield hybrid models for holistic risk prediction [34]. Emphasis on explainable AI will be crucial in demystifying “black-box” decisions, enabling clinicians to integrate outputs confidently into their workflows [35]. At the same time, regulatory pathways (the FDA's SaMD framework) evolve to expedite the safe deployment of these systems [36]. Future studies should investigate the cost-effectiveness of reducing unnecessary interventions and hospital readmissions, while also addressing ethical considerations, including bias mitigation and informed consent for AI-assisted diagnostics [37,38].

## 6 Conclusion

This systematic review and meta-analysis demonstrate that machine learning models, encompassing both traditional and deep learning algorithms, exhibit good diagnostic performance in assessing mitral regurgitation severity, highlighting the potential of ML to enhance diagnostic precision, reduce inter-observer variability, and support clinical decision-making in MR management. However, substantial heterogeneity exists across studies, driven by variations in validation approaches and patient populations, as well as the predominance of retrospective designs and limited external validation, which temper the generalizability of these results. The moderate certainty of evidence, as assessed by GRADE, underscores the need for caution in extrapolating findings to diverse clinical settings.

## CRediT authorship contribution statement

**Pooya Eini:** Writing – review & editing, Writing – original draft, Visualization, Validation, Supervision, Software, Resources, Project administration, Methodology, Investigation, Formal analysis, Data curation, Conceptualization. **Golnaz Houshmand:** Writing – review & editing, Writing – original draft, Validation, Supervision, Methodology. **Homa serpoush:** Writing – review & editing, Writing – original draft, Formal analysis, Data curation. **Mohammad Rezayee:** Writing – original draft, Visualization, Validation, Software, Resources. **Milan Kassulke:** Software, Resources, Methodology, Investigation.

## Availability of data

All data are included in the manuscript and supplementary files.

## Clinical trial number

Not applicable.

## Ethics approval and consent to participate

Not applicable.

## Consent for publication

Not applicable.

## Generative AI

In preparing this article, the authors utilized the Grammarly application to enhance linguistic accuracy and clarity. The manuscript underwent meticulous double-checking to ensure precision, and the authors assume full responsibility for the integrity and originality of the content presented herein.

## Funding

Not applicable.

## Competing interests

The authors declare that they have no competing interests.

## References

[1] Vahanian A., Beyersdorf F., Praz F. (2022). 2021 ESC/EACTS guidelines for the management of valvular heart disease: developed by the task force for the management of valvular heart disease of the european society of cardiology (ESC) and the european association for cardio-thoracic surgery (EACTS). Eur. Heart J..

[2] Coffey S., Roberts-Thomson R., Brown A. (2021). Global epidemiology of valvular heart disease. Nat. Rev. Cardiol..

[3] Goel S.S., Bajaj N., Aggarwal B. (2014). Prevalence and outcomes of unoperated patients with severe symptomatic mitral regurgitation and heart failure: comprehensive analysis to determine the potential role of MitraClip for this unmet need. J. Am. Coll. Cardiol..

[4] d'Arcy J.L., Coffey S., Loudon M.A. (2016). Large-scale community echocardiographic screening reveals a major burden of undiagnosed valvular heart disease in older people: the OxVALVE population cohort study. Eur. Heart J..

[5] Parcha V., Patel N., Kalra R., Suri S.S., Arora G., Arora P. (2020). Mortality due to mitral regurgitation among adults in the United States: 1999-2018. Mayo Clin. Proc..

[6] Dziadzko V., Clavel M.A., Dziadzko M. (2018). Outcome and undertreatment of mitral regurgitation: a community cohort study. Lancet.

[7] Enriquez-Sarano M., Akins C.W., Vahanian A. (2009). Mitral regurgitation. Lancet.

[8] Praz F., Borger M.A., Lanz J. (2025). 2025 ESC/EACTS guidelines for the management of valvular heart disease: developed by the task force for the management of valvular heart disease of the european society of cardiology (ESC) and the European association for cardio-thoracic surgery (EACTS). Eur. Heart J..

[9] Zoghbi W.A., Adams D., Bonow R.O. (2017). Recommendations for noninvasive evaluation of native valvular regurgitation: a report from the American society of echocardiography developed in collaboration with the society for cardiovascular magnetic resonance. J. Am. Soc. Echocardiogr..

[10] Lim D.S., Smith R.L., Gillam L.D. (2022). Randomized comparison of transcatheter edge-to-edge repair for degenerative mitral regurgitation in prohibitive surgical risk patients. JACC Cardiovasc. Interv..

[11] Eini P., Eini P., Serpoush H., Rezayee M. (2025). Diagnostic performance of machine learning algorithms for predicting heart failure in diabetic patients: a systematic review and meta-analysis. Endocrinology, Diabetes & Metabolism.

[12] Eini P., Eini P., serpoush H., Rezayee M., Tremblay J. (2025). Machine learning models for carotid artery plaque detection: a systematic review of ultrasound-based diagnostic performance. J. Stroke Cerebrovasc. Dis..

[13] Hassani Ahangar M., Aghazadeh-Habashi K., Rahi A. (2025). The significance of S100β and neuron-specific enolase (NSE) in postoperative cognitive dysfunction following cardiac surgery: a systematic review and meta-analysis. Eur. J. Med. Res..

[14] Sermesant M., Delingette H., Cochet H., Jaïs P., Ayache N. (2021). Applications of artificial intelligence in cardiovascular imaging. Nat. Rev. Cardiol..

[15] Narang A., Bae R., Hong H. (2021). Utility of a deep-learning algorithm to guide novices to acquire echocardiograms for limited diagnostic use. JAMA Cardiol..

[16] Yuan N., Jain I., Rattehalli N. (2021). Systematic quantification of sources of variation in ejection fraction calculation using deep learning. JACC, Cardiovasc. Imaging.

[17] Sveric K.M., Ulbrich S., Dindane Z. (2024). Improved assessment of left ventricular ejection fraction using artificial intelligence in echocardiography: a comparative analysis with cardiac magnetic resonance imaging. Int. J. Cardiol..

[18] Dey D., Slomka P.J., Leeson P. (2019). Artificial intelligence in cardiovascular imaging: JACC state-of-the-art review. J. Am. Coll. Cardiol..

[19] Østvik A., Smistad E., Aase S.A., Haugen B.O., Lovstakken L. (2019). Real-time standard view classification in transthoracic echocardiography using convolutional neural networks. Ultrasound Med. Biol..

[20] Page M.J., McKenzie J.E., Bossuyt P.M. (2021). The PRISMA 2020 statement: an updated guideline for reporting systematic reviews. Br. Med. J..

[21] Moons K.G.M., Damen J.A.A., Kaul T. (2025). PROBAST+AI: an updated quality, risk of bias, and applicability assessment tool for prediction models using regression or artificial intelligence methods. Br. Med. J..

[22] Moghaddasi H., Nourian S. (2016). Automatic assessment of mitral regurgitation severity based on extensive textural features on 2D echocardiography videos. Comput. Biol. Med..

[23] Balodi A., Anand R.S., Dewal M.L., Rawat A. (2020). Severity analysis of mitral regurgitation using discrete wavelet transform. IETE J. Res..

[24] Zhang Q., Liu Y., Mi J. (2021). Automatic assessment of mitral regurgitation severity using the mask R-CNN algorithm with color doppler echocardiography images. Comput. Math. Methods Med..

[25] Yang F., Zhu J., Wang J. (2022). Self-supervised learning assisted diagnosis for mitral regurgitation severity classification based on color doppler echocardiography. Ann. Transl. Med..

[26] Bernard J., Yanamala N., Shah R. (2023). Integrating echocardiography parameters with explainable artificial intelligence for data-driven clustering of primary mitral regurgitation phenotypes. JACC, Cardiovasc. Imaging.

[27] Long A., Haggerty C.M., Finer J. (2024). Deep learning for echo analysis, tracking, and evaluation of mitral regurgitation (DELINEATE-MR). Circulation.

[28] Zhong L., Deng Q., Wang Y. (2024). A fully convolutional neural network for the quantification of mitral regurgitation in echocardiography. Quant. Imag. Med. Surg..

[29] Sadeghpour A., Jiang Z., Hummel Y.M. (2025). An automated machine learning–based quantitative multiparametric approach for mitral regurgitation severity grading. JACC, Cardiovasc. Imaging.

[30] Long A., Finer J., Hartman H. (2025). Deep learning for echocardiographic assessment and risk stratification of aortic, mitral, and tricuspid regurgitation: the DELINEATE-regurgitation study. Eur. Heart J..

[31] Penso M., Pepi M., Mantegazza V. (2021). Machine learning prediction models for mitral valve repairability and mitral regurgitation recurrence in patients undergoing surgical mitral valve repair. Bioengineering.

[32] Chang C.C., Hsieh M.T., Lee Y.H. (2025). Deep learning model for identifying significant tricuspid regurgitation using standard 12-lead electrocardiogram. International Journal of Cardiology Cardiovascular Risk and Prevention.

[33] Chen J., Chen Y., Huang S. (2026). The association between liver disease and stroke risk: a cross-sectional study with machine learning in a large-scale Chinese cohort. International Journal of Cardiology Cardiovascular Risk and Prevention.

[34] Cui Y.Y., Zhang Y., Zeng L. (2025). Machine learning and Mendelian randomization identify key lifestyle factors in coronary heart disease: a NHANES-based study. International Journal of Cardiology Cardiovascular Risk and Prevention.

[35] Kasim S., Amir Rudin P.N.F., Kiew X.N. (2025). Enhancing cardiovascular risk prediction in Asian populations: a machine learning approach integrated with digital health platforms. International Journal of Cardiology Cardiovascular Risk and Prevention.

[36] Wu J., Huang D., Li J., Yi J., Lei Y., Yin J. (2025). Predicting cardiovascular disease and all-cause mortality using the lymphocyte-to-monocyte ratio: insights from explainable machine learning models. International Journal of Cardiology Cardiovascular Risk and Prevention.

[37] Yuting Y., Shan D. (2025). Associations between urinary and blood heavy metal exposure and heart failure in elderly adults: insights from an interpretable machine learning model based on NHANES (2003-2020). International Journal of Cardiology Cardiovascular Risk and Prevention.

[38] Eini P., Eini P., Serpoush H., Rezayee M., Tremblay J. (2025). Advancing mortality prediction in pulmonary embolism using machine learning algorithms-systematic review and meta-analysis. Pulm. Circ..

