# Supplementary material for: Accuracy of machine learning models for mitral regurgitation severity assessment: A systematic review and meta-analysis
Source: Int J Cardiol Cardiovasc Risk Prev. 2026 Apr 22;29:200642. doi: 10.1016/j.ijcrp.2026.200642 (PMC13129465; doi:10.1016/j.ijcrp.2026.200642)
Supplement: Multimedia component 1 [file mmc1.docx]

| Supplementary Table 1. Search syntax for different databases. | | |
| --- | --- | --- |
| Database | Syntax | Results |
| Pubmed | ("Insufficiency, Mitral Valve" OR "Valve Insufficiency, Mitral" OR "Mitral Incompetence" OR "Incompetence, Mitral" OR "Mitral Insufficiency" OR "Insufficiency, Mitral" OR "Mitral Regurgitation" OR "Regurgitation, Mitral" OR "Mitral Valve Incompetence" OR "Incompetence, Mitral Valve" OR "Valve Incompetence, Mitral" OR "Mitral Valve Regurgitation" OR "Regurgitation, Mitral Valve" OR "Valve Regurgitation, Mitral") AND ("Artificial Intelligence"[MeSH Terms] OR "Artificial Intelligence" OR "Artificial Intelligence Network" OR "Artificial Intelligence Networks" OR "Machine Intelligence" OR "Machine Learning" OR "Deep Learning" OR "Convolutional Neural Network") | 294 |
| WEB OF SCIENCE | TS=("Insufficiency, Mitral Valve" OR "Valve Insufficiency, Mitral" OR "Mitral Incompetence" OR "Incompetence, Mitral" OR "Mitral Insufficiency" OR "Insufficiency, Mitral" OR "Mitral Regurgitation" OR "Regurgitation, Mitral" OR "Mitral Valve Incompetence" OR "Incompetence, Mitral Valve" OR "Valve Incompetence, Mitral" OR "Mitral Valve Regurgitation" OR "Regurgitation, Mitral Valve" OR "Valve Regurgitation, Mitral") AND TS=("Artificial Intelligence" OR "Artificial Intelligence Network" OR "Artificial Intelligence Networks" OR "Machine Intelligence" OR "Machine Learning" OR "Deep Learning" OR "Convolutional Neural Network") | 324 |
| Scopus | TITLE-ABS-KEY("Insufficiency, Mitral Valve" OR "Valve Insufficiency, Mitral" OR "Mitral Incompetence" OR "Incompetence, Mitral" OR "Mitral Insufficiency" OR "Insufficiency, Mitral" OR "Mitral Regurgitation" OR "Regurgitation, Mitral" OR "Mitral Valve Incompetence" OR "Incompetence, Mitral Valve" OR "Valve Incompetence, Mitral" OR "Mitral Valve Regurgitation" OR "Regurgitation, Mitral Valve" OR "Valve Regurgitation, Mitral") AND TITLE-ABS-KEY("Artificial Intelligence" OR "Artificial Intelligence Network" OR "Artificial Intelligence Networks" OR "Machine Intelligence" OR "Machine Learning" OR "Deep Learning" OR "Convolutional Neural Network") | 402 |
| EMBASE | ('Insufficiency, Mitral Valve':ti,ab,kw OR 'Valve Insufficiency, Mitral':ti,ab,kw OR 'Mitral Incompetence':ti,ab,kw OR 'Incompetence, Mitral':ti,ab,kw OR 'Mitral Insufficiency':ti,ab,kw OR 'Insufficiency, Mitral':ti,ab,kw OR 'Mitral Regurgitation':ti,ab,kw OR 'Regurgitation, Mitral':ti,ab,kw OR 'Mitral Valve Incompetence':ti,ab,kw OR 'Incompetence, Mitral Valve':ti,ab,kw OR 'Valve Incompetence, Mitral':ti,ab,kw OR 'Mitral Valve Regurgitation':ti,ab,kw OR 'Regurgitation, Mitral Valve':ti,ab,kw OR 'Valve Regurgitation, Mitral':ti,ab,kw) AND ('Artificial Intelligence':ti,ab,kw OR 'Artificial Intelligence Network':ti,ab,kw OR 'Artificial Intelligence Networks':ti,ab,kw OR 'Machine Intelligence':ti,ab,kw OR 'Machine Learning':ti,ab,kw OR 'Deep Learning':ti,ab,kw OR 'Convolutional Neural Network':ti,ab,kw) | 208 |
| EBSCO | TI,AB("Insufficiency, Mitral Valve" OR "Valve Insufficiency, Mitral" OR "Mitral Incompetence" OR "Incompetence, Mitral" OR "Mitral Insufficiency" OR "Insufficiency, Mitral" OR "Mitral Regurgitation" OR "Regurgitation, Mitral" OR "Mitral Valve Incompetence" OR "Incompetence, Mitral Valve" OR "Valve Incompetence, Mitral" OR "Mitral Valve Regurgitation" OR "Regurgitation, Mitral Valve" OR "Valve Regurgitation, Mitral") AND TI,AB("Artificial Intelligence" OR "Artificial Intelligence Network" OR "Artificial Intelligence Networks" OR "Machine Intelligence" OR "Machine Learning" OR "Deep Learning" OR "Convolutional Neural Network") | 73 |
| Total |  | 1301 |

| 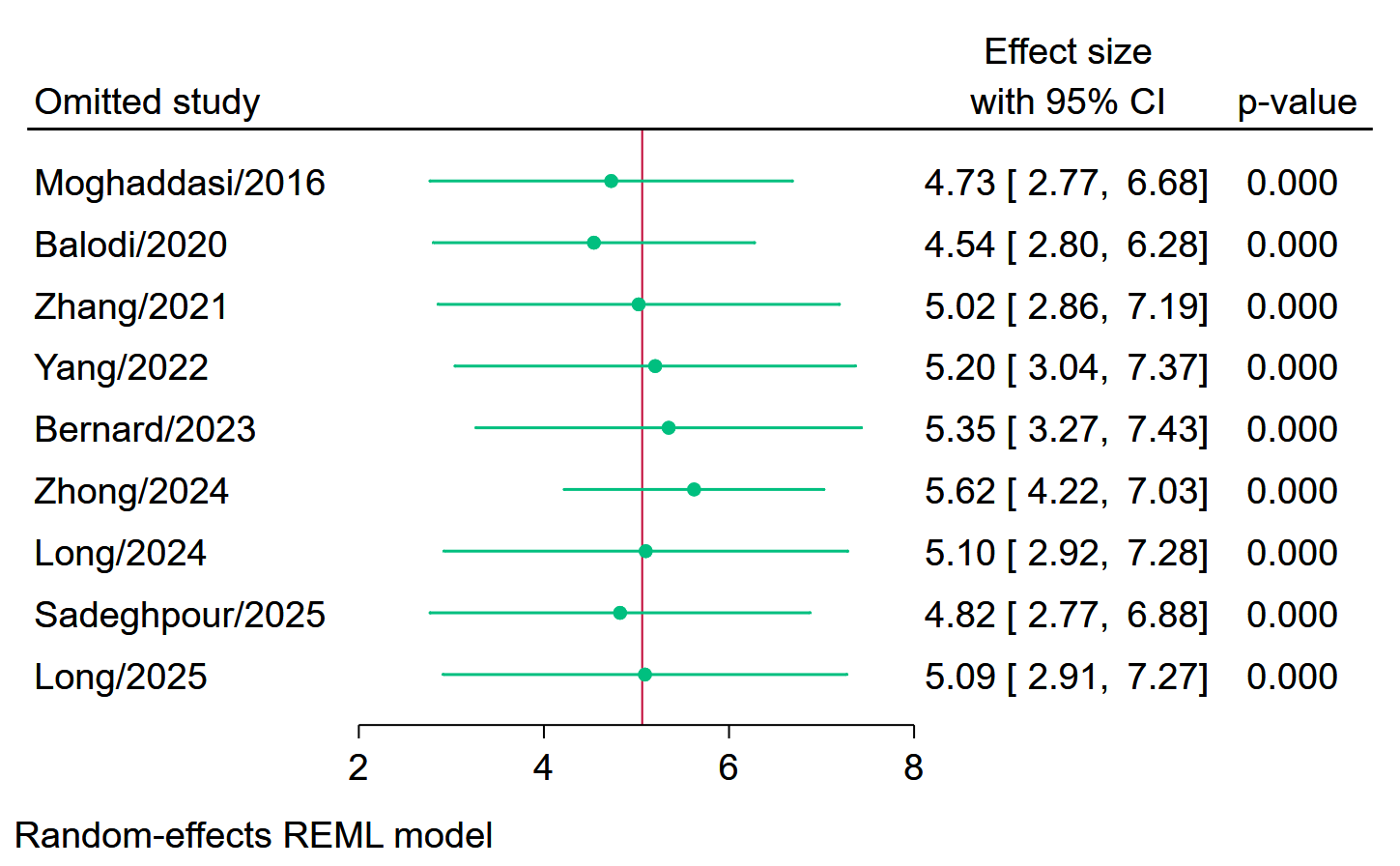 |
| --- |
| supplementary Figure 1. Leave-One-Out analysis. |

| **Supplementary Table 2. Meta-regression results for potential sources of heterogeneity** | | | | |
| --- | --- | --- | --- | --- |
| **Parameter** | **LRTChi²** | **P value** | **I²** | **I² lo** |
| Model type | 5.09 | 0.08 | 61 | 11 |
| Validation group | 7.99 | 0.02 | 75 | 45 |
| Log sample size | 14.00 | <0.001 | 86 | 70 |
| Country | 0.73 | 0.69 | 0 | 0 |

| 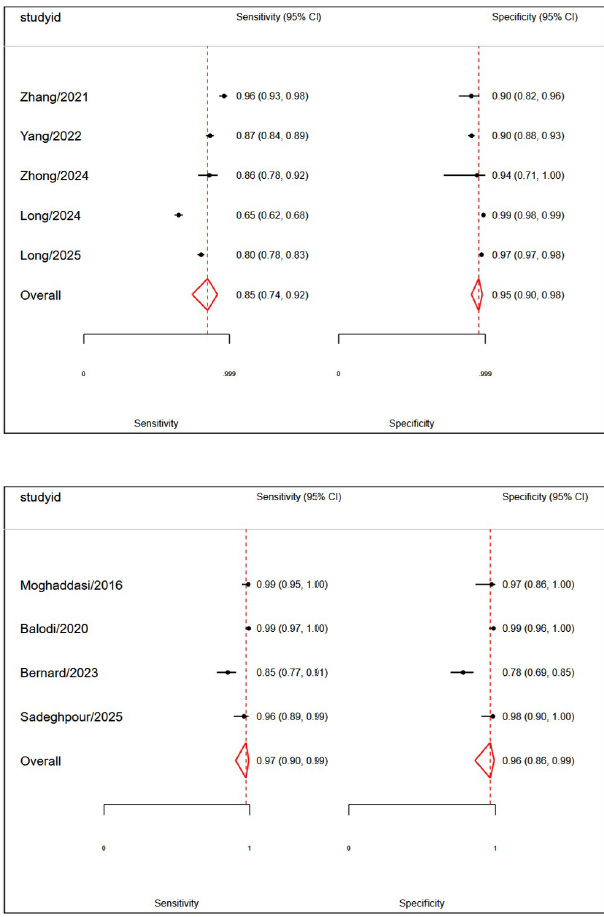 |
| --- |
| Supplementary figure 2. Subgroup analysis based on algorithm type. |
| 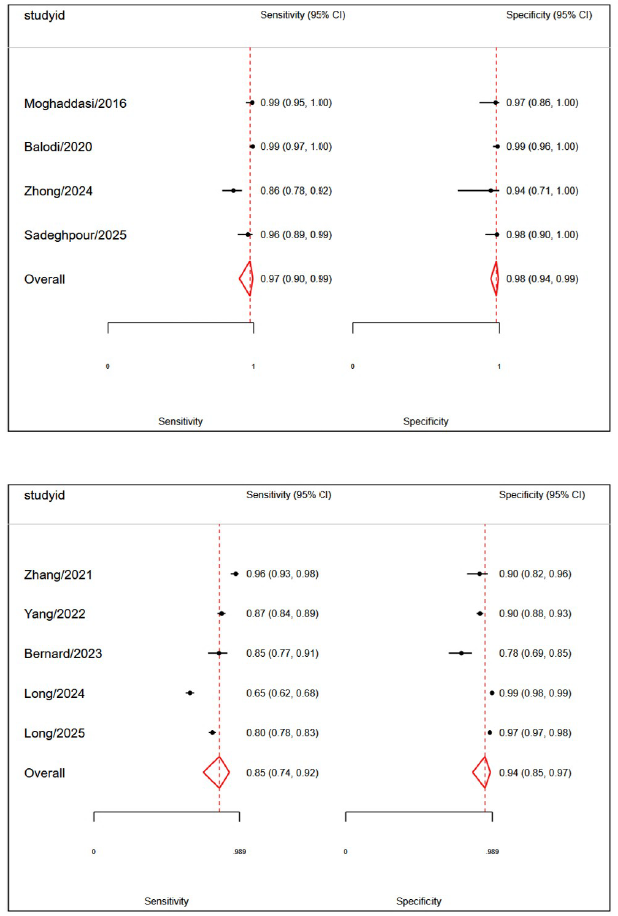 |
| Supplementary figure 3. Subgroup analysis based on frames versus full video‑based echocardiographic inputs. |
| 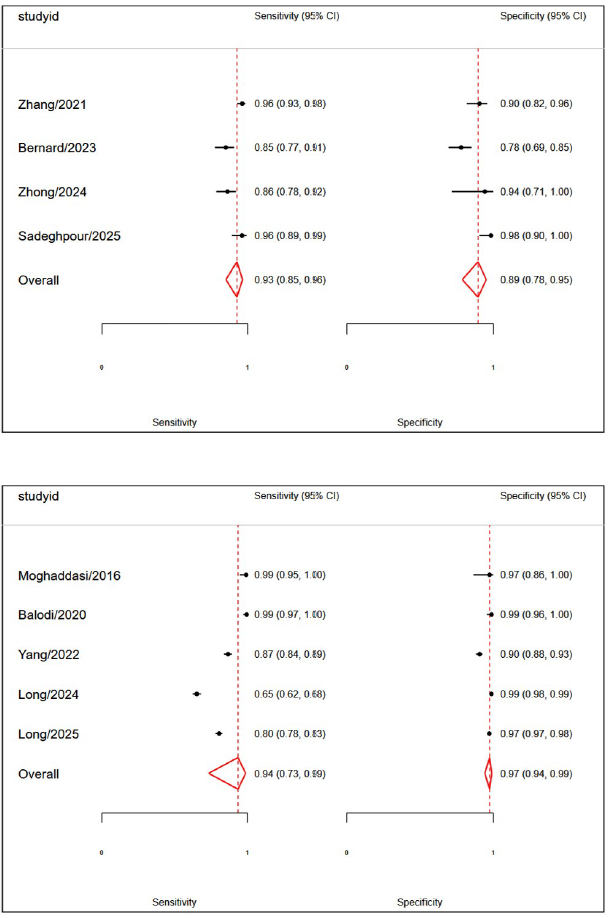 |
| Supplementary figure 4. Subgroup analysis based on MR assessment strategy. |
| 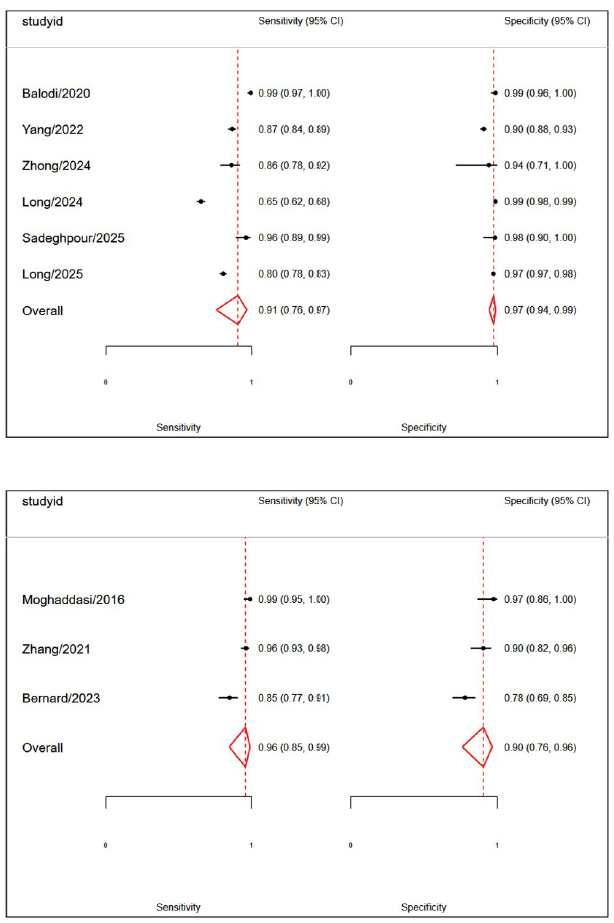 |
| Supplementary figure 5. Subgroup analysis based on human interpretation or ML‑based measurements. |

| Supplementary table 3. quality assessment check list based on PROBAT+AI tool. |
| --- |
| **Development (Dev) Domains**  **Participants (Dev)**   1. Were participants in the development dataset representative of the target population (e.g., patients with suspected or diagnosed mitral regurgitation across a range of severities), with clearly defined inclusion/exclusion criteria and sufficient sample size for AI model development?   **Predictors (Dev)** 2. Were predictors (e.g., echocardiographic features, clinical variables) clearly defined, consistently measured, and appropriately processed (e.g., normalization, handling of missing data) without knowledge of the outcome to avoid bias in model development?  **Outcomes (Dev)** 3. Was the outcome (mitral regurgitation severity, based on standardized echocardiographic or guideline-defined criteria) clearly defined and assessed independently of predictors to minimize misclassification bias?  **Analyses (Dev)** 4. Were AI model development methods (e.g., algorithm selection, cross-validation, hyperparameter tuning) appropriate, transparent, and free from data leakage or overfitting, with performance metrics reported alongside uncertainty measures?  **Overall (Dev)** 5. Does the development process align with the study question, ensuring the model’s applicability to the target population, predictors, and outcomes for MR severity assessment?  **Evaluation (Eval) Domains**  **Participants (Eval)** 6. Were participants in the evaluation dataset representative of the target population for MR severity assessment, with consistent inclusion/exclusion criteria and sufficient diversity to ensure robust performance assessment?  **Predictors (Eval)** 7. Were predictors in the evaluation dataset measured consistently with those in the development dataset, using appropriate preprocessing and without knowledge of the outcome, to ensure unbiased performance estimates?  **Outcomes (Eval)** 8. Was the outcome in the evaluation dataset defined and assessed using the same standardized MR severity criteria as in development, independently of predictors, to ensure valid performance evaluation?  **Analyses (Eval)** 9. Were evaluation methods (e.g., external validation, performance metrics such as sensitivity, specificity, AUC) appropriate, independent of development data, and reported with uncertainty measures?  **Overall (Eval)** 10. Does the evaluation process align with the study question, ensuring the model’s performance is applicable to the target population, predictors, and outcomes for MR severity assessment?  **Applicability (App) Domains**  **Participants (App)** 11. Does the study’s participant population (e.g., age, sex, clinical setting) match the intended target population for MR severity assessment in clinical practice?  **Predictors (App)** 12. Do the study’s predictors (e.g., echocardiographic imaging features, clinical variables) align with those available in routine practice settings, ensuring feasibility of MR severity assessment in real-world contexts?  **Outcomes (App)** 13. Does the study’s outcome definition align with established clinical standards and echocardiographic guidelines for MR severity assessment?  **Analyses (App)** 14. Do the study’s analysis methods (e.g., diagnostic accuracy metrics, subgroup analyses) support clinical decision-making needs for MR severity assessment in diverse settings?  **Overall (App)** 15. Does the study’s design and findings align with the study question, ensuring applicability to the target population, predictors, outcomes, and clinical contexts for MR severity assessment? |
